# Post-translational modifications (PTM): analytical approaches, signaling, physiology and pathophysiology—part I

**DOI:** 10.1007/s00726-021-02984-y

**Published:** 2021-04-30

**Authors:** Dimitrios Tsikas

**Affiliations:** Core Unit Proteomics, Institute of Toxicology, Hannover Medical School, Carl-Neuberg-Str. 1, 30625 Hanover, Germany

**Fig. 1:**
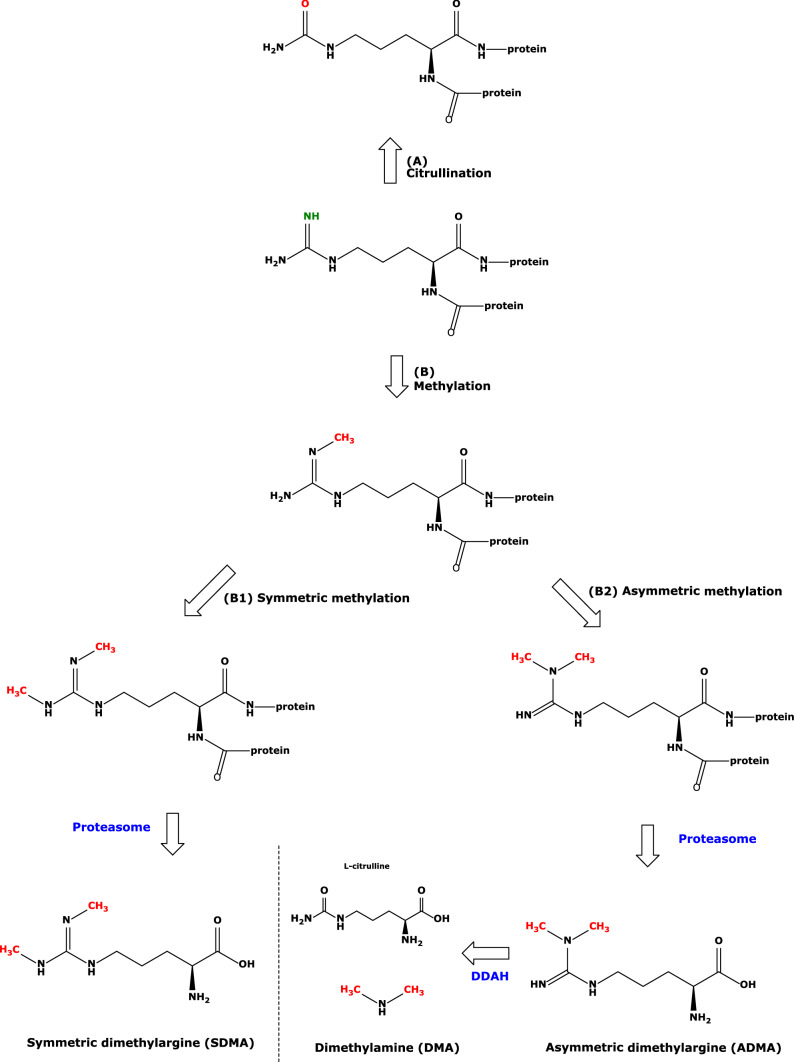
Simplified schematic of two major enzymatic post-translational modifications of l-arginine residues in proteins. **a** Citrullination catalyzed by protein-arginine deaminase (PAD). **b** *N*-Guanidine methylation and subsequent symmetric (B1) and asymmetric (B2) dimethylation catalyzed by protein-arginine methyl transferase (PRMT). Proteolysis releases the free amino acids monomethylarginine (not shown), symmetric dimethylarginine (SDMA) and asymmetric dimethylarginine (ADMA). ADMA is hydrolyzed to dimethylamine (DMA) and l-citrulline by dimethylarginine dimethylaminohydrolase (DDAH)

After their birth in ribosomes, the virgin and immaculate proteins undergo numerous modifications on their building blocks, the amino acids, both by chemical and enzymatic reactions. These so-called post-translational modifications (PTMs) do not only change the physicochemical properties including charge and solubility of proteins, but they have far-reaching consequences for health, disease, and even death in living organisms. Reportedly, the first scientific publications appeared as early as the 1940s and evolved drastically over the last two decades, indicating the strong interest of researchers from many different disciplines. With 6604 and 6139 articles, acetylation and methylation belong to the most common investigated PTMs, respectively. Amino acid residues such as arginine and lysine undergo many different PTMs. For example, the terminal guanidine (*N*^G^) group of arginine in proteins can be methylated by protein-arginine methyl-transferase (PRMT) and citrullinated by protein-arginine deiminase (PAD) (Fig. 1). Citrullination is considered to give rise to new antigenic epitopes leading to the generation of auto-antibodies and to play a particular role in rheumatoid disease (Catrina et al. 2021). Although known for many decades, the significance of *N*^G^-methylated proteins remains still to be explored in depth. Better understood is the biochemistry, biology, metabolism, and pharmacology of low-molecular-mass *N*^G^-methylated arginine metabolites. Proteolysis of *N*^G^-methylated proteins releases monomethylated Arg (MMA), symmetric dimethylarginine (SDMA) and asymmetric dimethylarginine (ADMA), with ADMA being hydrolyzed to its major metabolites dimethylamine (DMA) and l-citrulline (Fig. 1). ADMA and to a much lesser extent SDMA are inhibitors of the activity of nitric oxide synthase (NOS), but may also exert detrimental effects by other unknown mechanisms. Advanced glycation end-products (AGEs) belong to chemical PTMs (Nagai et al. 2014). These examples and the low-molecular-mass bioactive AGEs indicate that PTM does not only modify proteins, but may also serve as a source for biologically active metabolites and biomarkers, such as HbA_1c_, one of the earliest indices of diabetic control (Bunn 1981).

This volume presents the first part of a special issue of *Amino Acids*, a journal dedicated to amino acid, peptide and protein research, on PTMs including two review articles and five original research papers.

Samuel and colleagues provide an up-to-date review of the literature on mechanisms for PRMT regulation by endogenous modulators and synthetic inhibitors (Samuel et al. 2021). Sirover’s review highlights the role of several PTMs including acetylation and nitrosylation in the structure and function of the moonlighting GADPH including gene regulation (Sirover 2021).

Two original articles report on novel results on *N*-glycans as obtained by using UHPLC-MS/MS (Jinesh et al. 2021) and on peptide tyrosine nitration using spectroscopic methods (Niederhafner et al. 2021). The whole-body asymmetric arginine dimethylation was investigated in adult renal transplant recipients by measuring DMA, the major metabolite of ADMA, in urine by GC–MS (Post et al. 2021). This study revealed that the whole-body asymmetric arginine dimethylation is associated with all-cause mortality in adult renal transplant recipients and supports the particular role of this PTM in chronic kidney disease (Frenay et al. 2015; Said et al. 2019a, b).

*N*^ε^-Methylation of Lys residues in proteins generates mono-, di- and tri-methylated Lys proteins, of which the proteolysis yields the low-molecurar-mass monomethyllysine (MML), dimethyllysine (DML) and trimethyllysine (TML) derivatives. The significance of TML as a vascular risk factor was investigated in a clinical study on acute ischemic stroke using LC–MS/MS (Schwedhelm et al. 2021). This study observed that TML was not associated with incident MACE (stroke, myocardial infarction, death) and suggested that TML may play a different role in acute ischemic stroke compared with coronary artery disease patients. Potential differences between the methylation of arginine and lysine residues in proteins regarding various aspects including the biological activity of the Arg- and Lys-methylated proteins and their low-molecular-mass MMA, ADMA, SDMA, MML, DML, and TML derivatives remain to be investigated.

Like tyrosine nitration in peptides and proteins, *S*-transnitrosylation of cysteine residues in proteins is a less abundant but not less important PTM. *S*-Nitrosoalbumin is one of the most abundant endogenous *S*-nitrosylated proteins in human plasma and may serve as a carrier and source of NO-related bioactivity in human circulation including platelets and red blood cells. In an in vitro study, extracellular low-molecular-mass thiols including l-cysteine were found to mediate the inhibitory action of synthetic *S*-nitrosoalbumin on human platelet aggregation via *S*-transnitrosylation of the platelet surface proteins (Tsikas 2021).

We thank the authors for their contributions and the reviewers for their honorary engagement.
